# PRIMARY MOTIVATIONS OF AFRICAN AMERICANS AND LATINOS TO PARTICIPATE IN ALZHEIMER’S PREVENTION RESEARCH

**DOI:** 10.1093/geroni/igad104.3082

**Published:** 2023-12-21

**Authors:** Lizbeth Vera Murillo, Angel Collie, Maria Vander Meulen, Monique Villamor, Jody Nicholson-Bell

**Affiliations:** University of North Florida, Jacksonville, Florida, United States; University of North Florida, Jacksonville, Florida, United States; University of North Florida, Jacksonville, Florida, United States; University of North Florida, Jacksonville, Florida, United States; Clemson University, Clemson, South Carolina, United States

## Abstract

The prevalence of Alzheimer’s Disease and related dementias (ADRD) is higher among racial/ethnic minorities as compared to White/Caucasian individuals, yet there is poor representation in ADRD research for these minoritized groups. It is essential to investigate the motivation to participate in research for individuals from minoritized groups to optimize inclusiveness through recruitment and retention strategies. The current project explores racial/ethnic differences in motivations to participate in an ADRD prevention study using case-controlled matching between White/Caucasians (n=210; M = 71.01 years, SD = 4.41) and African American/Black (n=210; M = 71.26 years, SD = 4.47) participants, and between non-Hispanics (n = 158; M =71.13, SD = 5.33), and Hispanic participants (n = 157; M = 71.48 years, SD = 5.14). Results indicated Latinos/Hispanics were more likely to report concerns about brain health and aging (59.7%; χ ² = 3.99, p < .05, φ=.11) and improvement of personal brain health (60.9%; χ ² = 4.30, p < .05, φ=.12) as a motivator compared to non-Hispanics. African American/Blacks were more likely to report concerns about brain health (59.6%; χ ² = 4.66, p < .05, φ=.12) as a motivator compared to White/Caucasians. Data from the current study about the concerns of cognitive decline and brain health for African Americans/Blacks and Latinos/Hispanic opens an avenue by which participation in clinical trials may be increased by better project marketing/advertisement focusing on the advantages of preventative health behaviors involving non-pharmacological methods and cognitive health resources that might be available within a research environment.

